# Single‐Cell RNA Sequencing and Spatial Transcriptomics Reveal Pathogenesis of Meningeal Lymphatic Dysfunction after Experimental Subarachnoid Hemorrhage

**DOI:** 10.1002/advs.202301428

**Published:** 2023-05-21

**Authors:** Xiaoyu Wang, Anke Zhang, Qian Yu, Zelin Wang, Junjie Wang, Penglei Xu, Yibo Liu, Jianan Lu, Jingwei Zheng, Huaming Li, Yangjian Qi, Jiahao Zhang, Yuanjian Fang, Shenbin Xu, Jingyi Zhou, Kaikai Wang, Sheng Chen, Jianmin Zhang

**Affiliations:** ^1^ Department of Neurosurgery The Second Affiliated Hospital School of Medicine Zhejiang University Hangzhou Zhejiang 310003 China; ^2^ Clinical Research Center for Neurological Diseases of Zhejiang Province Hangzhou 310003 China; ^3^ Bioinformatics Shuzhi Biotech LLC Guangzhou 510805 China; ^4^ Department of Neurosurgery The Fourth Affiliated Hospital International Institutes of Medicine Zhejiang University School of Medicine Yiwu 322000 China; ^5^ Department of Gastrointestinal Surgery the Second Affiliated Hospital of Zhejiang University School of Medicine Hangzhou 310003 China; ^6^ Brain Research Institute Zhejiang University Hangzhou Zhejiang 310003 China; ^7^ MOE Frontier Science Center for Brain Science & Brain‐Machine Integration Zhejiang University Hangzhou Zhejiang 310003 P. R. China

**Keywords:** meningeal lymphatic, meningeal lymphatic endothelial cells, single‐cell RNA sequencing, spatial transcriptome, subarachnoid hemorrhage

## Abstract

Subarachnoid hemorrhage (SAH) is a devastating subtype of stroke with high mortality and disability rate. Meningeal lymphatic vessels (mLVs) are a newly discovered intracranial fluid transport system and are proven to drain extravasated erythrocytes from cerebrospinal fluid into deep cervical lymph nodes after SAH. However, many studies have reported that the structure and function of mLVs are injured in several central nervous system diseases. Whether SAH can cause mLVs injury and the underlying mechanism remain unclear. Herein, single‐cell RNA sequencing and spatial transcriptomics are applied, along with in vivo*/*vitro experiments, to investigate the alteration of the cellular, molecular, and spatial pattern of mLVs after SAH. First, it is demonstrated that SAH induces mLVs impairment. Then, through bioinformatic analysis of sequencing data, it is discovered that thrombospondin 1 (THBS1) and S100A6 are strongly associated with SAH outcome. Furthermore, the THBS1‐CD47 ligand‐receptor pair is found to function as a key role in meningeal lymphatic endothelial cell apoptosis via regulating STAT3/Bcl‐2 signaling. The results illustrate a landscape of injured mLVs after SAH for the first time and provide a potential therapeutic strategy for SAH based on mLVs protection by disrupting THBS1 and CD47 interaction.

## Graphical abstract

This study integrates single‐cell RNA sequencing and spatial transcriptomics to comprehensively characterize the composition and alterations of different cell clusters in meningeal lymphatic vessels (mLVs) tissues after subarachnoid hemorrhage (SAH) in mice models for the first time. Thrombospondin 1‐CD47 ligand‐receptor interaction is proven to induce meningeal lymphatic endothelial cell apoptosis, which might be the initial reason for mLVs injury after SAH.

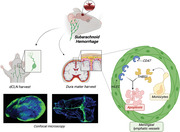

## 1 Introduction

Subarachnoid hemorrhage (SAH) is a devastating subtype of stroke, characterized by a high rate of disability and mortality.^[^ ^1^ ^]^ Early brain injury (EBI) and delayed cerebral ischemia (DCI) are the two major pathological processes after SAH. Regrettably, current therapies targeting both EBI and DCI show limited clinical benefits.^[^ ^2^ ^]^ Hence, there is an urgent need to develop novel therapeutic strategies to promote the neurological function recovery of SAH patients. Evidence has indicated that the extravasation of erythrocytes in cerebrospinal fluid (CSF) is crucial to the SAH progress, which severely disturbs the CSF circulation in both acute and late phases after SAH. CSF circulation dysfunction can directly lead to brain edema formation and hydrocephalus through increasing intracerebral pressure (ICP) and decreasing cerebral blood perfusion.^[^ ^3^ ^]^ Considering these SAH pathologies, accelerating extravasated blood clearance in subarachnoid space (SAS) may be an effective therapeutic approach.

Lymphatic drainage plays an important role in modulating tissue homeostasis, excess fluid clearance and macromolecules, and immune cell migration.^[^ ^4^ ^]^ The meningeal lymphatic system within the dura mater transports macromolecules away from the brain parenchyma and delivers CSF to the surrounding cervical lymph nodes.^[^ ^5^ ^]^ Weakening of this central nervous system (CNS) drainage system leads to impairment of pathological substance clearance and is associated with neurodegenerative and age‐associated neurological conditions.^[^ ^6^ ^]^ Subsequent studies have revealed that meningeal lymphatic vessels (mLVs) are responsible for the clearance of *β*‐amyloid and *α*‐synuclein, which lead to neurological deficits in Alzheimer's and Parkinson's diseases.^[^ ^7^ ^]^ Recently, mLVs have also been reported to drain extravasated erythrocytes from CSF into deep cervical lymph nodes (dCLNs) after SAH.^[^ ^3b^ ^]^ These findings further demonstrate that mLVs play key roles in the clearance of intracranial waste. However, little is known about the cellular architecture and underlying gene regulatory features of mLVs, especially after SAH.

Single‐cell RNA sequencing (scRNA‐Seq) makes it possible to identify cell subpopulations and define their unique function by exploring the unique transcriptomic profile of each single cell, which therefore has emerged as a powerful tool to further explain pathophysiological changes in diseases.^[^ ^8^ ^]^ However, information on spatial distribution and correlation with other cells of one individual cell is lost during tissue dissociation. Spatial transcriptomics (ST) is currently invented to identify cell types and cell‐to‐cell interactions on a structural and spatial level in a selected tissue, which can compensate for the drawbacks of scRNA‐seq.^[^ ^9^ ^]^ Integration of scRNA‐Seq with ST enables in situ reproduction and visualization of pathologies in injured tissue than scRNA‐Seq or ST alone,^[^ ^10^ ^]^ and has been applied in various tumor diseases.^[^ ^11^ ^]^ Therefore, by integrating these two techniques, we will be able to better understand mLVs pathophysiology after SAH.

In this study, we used scRNA‐seq and ST to comprehensively characterize the composition and alterations of different cell clusters in mLVs tissues after SAH in the mice model for the first time. After confirming that SAH could cause mLVs impairment, we then analyzed our sequencing data and discovered two significant factors, thrombospondin 1 (THBS1) and S100A6, were related to SAH prognosis. Finally, THBS1‐CD47 ligand‐receptor (L‐R) interaction was proved to induce meningeal lymphatic endothelial cells (mLECs) apoptosis through mediating STAT3/Bcl‐2 signaling, which might be the initial reason for mLVs injury after SAH.

## 2 Results

### 2.1 Single‐Cell Transcriptomic Atlas of mLVs Alterations at Different Time Courses after SAH

**Figure 1:**
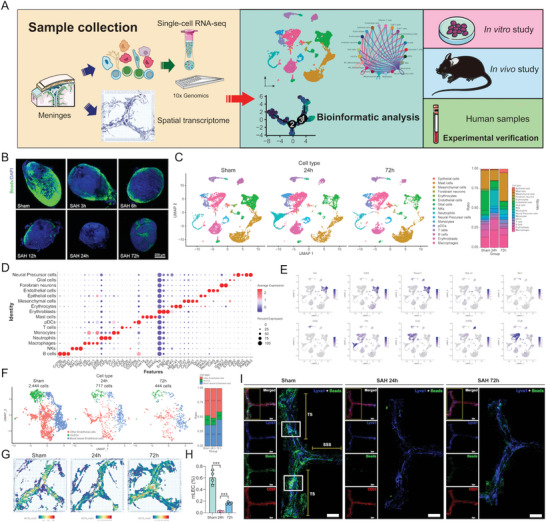
Single‐cell transcriptomic atlas of mLVs alterations at different time courses after SAH. A) Graphic overview of our study design. Mice dura matters were dissociated into single‐cell suspension and used for scRNA‐seq with 10× Genomics. Dura slides were processed to obtain spatial transcriptomics by 10× Genomics Visium. Integrated analysis of single‐cell transcriptome data and the following in vivo*/*vitro experiments were performed. B) Representative images of fluorescent beads accumulation in dCLNs at 3, 6, 12, 24, and 72 h post‐SAH. Scale bar: 200 µm. C) UMAP plots of 16 cell clusters with different colors (left) and bar charts of cluster proportion (right) in each group. D) Dot plots showing the average expression of known markers in 16 cell types. The dot size represents the percentage of cells expressing the indicated genes in each cell type. E) Expression levels of selected known marker genes across 25 536 unsorted cells illustrated in UMAP plots from both Sham and SAH dura in mice. F) High‐resolution UMAP plots of three re‐defined endothelial cell sub‐clusters in each group. G) Predicted spatial distribution of mLECs by RCTD. H) Flow cytometric analysis of mLECs percentage at different time courses after SAH, *n* = 4 per group, *** *p* < 0.001 versus Sham and 24 h by paired two‐tailed Student's *t*‐test. I) Representative confocal images of mLVs region at different time courses after SAH. An enlarged view of TS and SSS is shown on the right in each group. Solid boxes show the hotspots along the TS. Lyve1 (blue), Beads (green), and CD31 (red). Scale bar: 1000 µm.

To investigate whether mLVs are impaired after SAH, we used experimental SAH models by injecting autologous blood into the prechiasmatic cisternae. This model is ideal for exploring CNS lymphatic function because it does not demand complete exposure to neck anatomy, which can avoid direct impact on mLVs (Figure S1A, Supporting Information). Also, fluorescent beads were injected intra‐cisterna magna (i.c.m.) at various time courses after SAH modeling for further assessment of the drainage function of mLVs (Figure S1A, Supporting Information). The results presented a substantial decrease in beads drainage to the dCLNs as early as 3 h after SAH, and the drainage impairment remained until 72 h post‐injury (**Figure** **1**B and Figure S1B, Supporting Information). As for the upstream path of dCLNs, decreased drainage and wounded structure also presented in mLVs at 24 and 72 h after SAH (Figure 1I and Figure S1C, Supporting Information).

To comprehensively elucidate the cellular composition alteration of mLVs after SAH, we performed scRNA‐seq (10× Genomics) on mice in different groups (Sham group, SAH 24 h group, SAH 72 h group, ten mice per group) (Figure 1A). After excluding damaged or dead cells and putative cell doublets out of the original scRNA‐seq data and adjacent sequent analysis, a total of 25 536 cell transcriptomes of 30 mice were retained, in which 9891 cells were originated from the sham group, 8262 cells were from SAH 24 h group, and 7383 were cells from SAH 72 h group (Figure 1C). Then we normalized the gene expression referring to sequencing depth and mitochondrial read count and applied principal component analysis (PCA) based on highly variably expressed genes across cells. To correct the batch effect, we integrated scRNA‐seq data with the Seurat integration method.^[^ ^12^ ^]^ Further, a unified uniform manifold approximation and projection (UMAP) embedding space and graph‐based clustering approach was used to identify cell clusters. Clusters were annotated with well‐known markers. The cells were classified into 16 major cell types (Figure 1C–E) including B cells marked by *Cd79A*, *Cd79B*, and *Ms4a1*,^[^ ^13^ ^]^ NKs marked by *Ncr1*, *Klrb1c*, *and Nkg7*,^[^ ^14^ ^]^ macrophages marked by *C1qb*, *Cd68*, *Csf1r*, and *Mrc1*,^[^ ^15^ ^]^ neutrophils marked by *S100α9*, *IL1β*, and *Csf3r*,^[^ ^16^ ^]^ monocytes marked by Ccr2 and Ly6c2,^[^ ^17^ ^]^ T cells marked by *Cd3d*, *Cd3e*, and *Cd247*,^[^ ^18^ ^]^ pDCs marked by *Cd209a* and *Cd74*,^[^ ^19^ ^]^ mast cells marked by *Fcer1a*, *Kit*, and *Ms4a2*,^[^ ^20^ ^]^ erythroblasts marked by *Ttr*, *Enpp2*, and *Ptgds*, erythrocyte marked by *Car2*, *Hba‐a1*, and *Hbb‐bs*,^[^ ^21^ ^]^ mesenchymal cells marked by *Col1a1*, *Col1a2*, *Col3a1*, and *DCN*,^[^ ^13^ ^]^ epithelial cells identified by the expression of *Birc5* and *Ube2c*,^[^ ^22^ ^]^ endothelial cell marked by *Pecam1*, *Cdh5*, and *Kdr*,^[^ ^13^ ^]^ forebrain neuron marked by *Gng13* and *S100α5*,^[^ ^23^ ^]^ glial cell marked by *S100b* and *Cdh2*,^[^ ^24^ ^]^ and neuralprecursor cells marked by *Cdh2*, *Pde6g*, and *Gnb3*.^[^ ^24^ ^]^ Although all 16 major cell types were presented in mLVs both in sham and SAH groups (Figure 1C), the dynamic changes of each cell type were different. Meanwhile, given that mLECs originated from endothelial cells, we further performed clustering analysis for endothelial cells and obtained three sub‐clusters. This result showed that mLECs proportion became the least at 24 h after SAH (Figure 1F), indicating that at this time course, mLVs injury might be the severest.

Similarly, flow cytometric analysis showed the population of CD45^–^CD31^+^LYVE1^+^ cells (mLECs) declined after SAH, and that the most serious injury happened 24 h post SAH (Figure 1H). Moreover, injured mice got lower modified Garcia scores, prolonged *T*_turn_ and *T*_total,_ prolonged deficits, and delayed recovery compared to the sham group in several neurological function tests, respectively (Figure S1D, Supporting Information). Taken together, these results demonstrated that SAH caused the injury of mLVs structure and drainage function, and elicited loss of neurological function in mice in the acute phase.

### 2.2 Deconvolution of Spatial Atlas and Visualization of Cell Type Interactions

**Figure 2:**
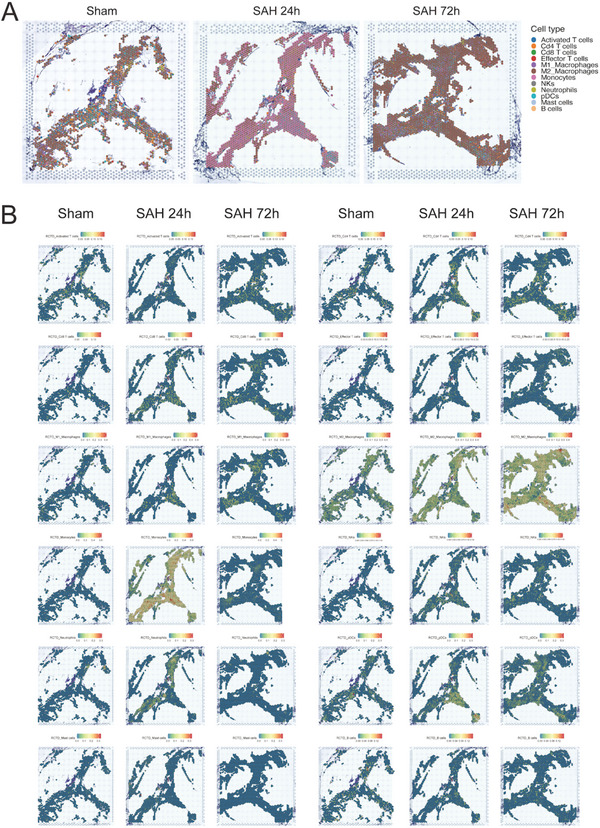
Deconvolution of spatial atlas of mLVs after SAH. A) Spatial scatter pie plots representing the proportion of different cell types from reference atlas within capture locations in mice dura at different time courses after SAH. B) Predicted spatial localization of immune cell types around mLVs by RCTD.

As mentioned above, scRNA‐seq data lost the spatial characteristics of each cell. Therefore, we then combined ST to improve the integrity of sequencing data and reveal the spatial distribution of all cells. After hematoxylin and eosin (H&E) staining and brightfield imaging, mLVs slides were subjected to discover distinct histological features. Standard quality control and dimensionality reduction were performed using Seurat methods, and visualization was realized through t‐distributed Stochastic Neighbor Embedding (T‐SNE). Based on differentially expressed genes (DEGs) in each cluster, stepwise cell clustering was performed to construct an ST map. As was mentioned in a recent study, we similarly used RCTD software to deconvolute each tissue‐covering spot,^[^ ^25^ ^]^ and further reconstructed the layered and segmented structure of sinus anatomy. The predicted localization of the 12 immune cell clusters confirmed their enrichment in specific regions (e.g., superior sagittal sinus [SSS], transverse sinus [TS], and hotspot). We mapped the ratios of each cell cluster within each spot as representative pie charts (scatter pies) both on the SSS and TS. Interestingly, distinct changes occurred in infiltrated immune cells into mLVs after injury. Compared to normal mLVs, infiltrated monocytes significantly increased and became the main group among all cell clusters 24 h after SAH. Although infiltrated macrophages were the most at 72 h with the majority subtype of M2, there were significantly more monocytes and M1 type macrophages gathering at the hotspot site (**Figure** **2**A).

To further explore the alterations in the meningeal immune microenvironment, we attempted to map the scRNA‐seq data of each cell to ST to accurately reveal the infiltration of immune cells into mLVs after SAH. Immune cell infiltration was dominated by monocytes at 24 h and by macrophages at 72 h after SAH. Neutrophil infiltration peaked at 24 h and returned to normal level (sham) by 72 h. T cell infiltration gradually increased at different time courses after injury while B cell infiltration was relatively stable. NKs and pDC infiltration also continuously increased after injury (Figure 2B). These results reflected the cellular changes of mLVs in different stages as SAH progressed.

### 2.3 Cell‐to‐Cell Communication Analysis Revealed THBS1‐CD47 Ligand‐Receptor Pair Activation Associated with mLVs Impairment

**Figure 3:**
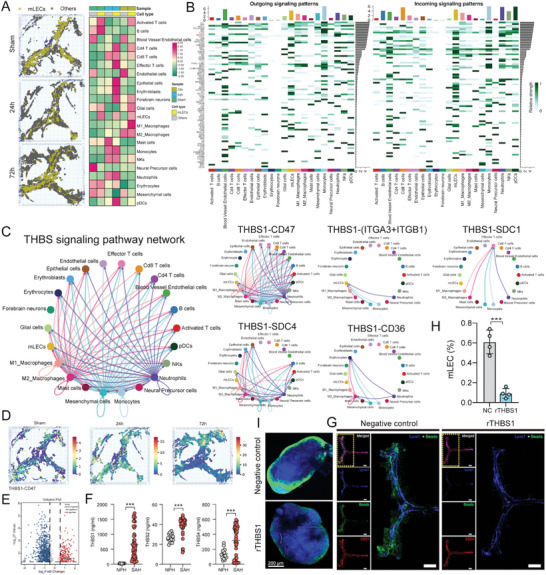
Intercellular communication networks in and around mLVs. A) Spatial visualization of immune cell infiltration based on mLECs distribution. B) Outgoing and incoming signal patterns among all meningeal cells. C) THBS signaling pathway network among all cell types. D) Spatial visualization of THBS1‐CD47 L‐R pair distribution by stLearn. E) Volcano plots showing that THBS1 is one of the upregulated DEGs in the SAH group compared to the NPH group identified by mass spectrometry. F) Quantification of THBS1, THBS2, and THBS4 expression in CSF samples from SAH patients compared to those from NPH patients by ELISA assay, *** *p* < 0.001 versus NPH by paired two‐tailed Student's *t*‐test. G) Representative confocal images of mLVs region of the negative control (NC) group and recombinant THBS1 protein treatment (rTHBS1) group. An enlarged view of TS and SSS is shown on the right in each group. Lyve1 (blue), Beads (green), and CD31 (red). Scale bar: 1000 µm. H) Flow cytometric analysis of mLECs percentage in NC group and rTHBS1 group, *n* = 4 per group, *** *p* < 0.001 versus NC by paired two‐tailed Student's *t*‐test. I) Representative images of beads accumulation in dCLNs of the NC group and rTHBS1 group. Scale bar: 200 µm.

Given that immune inflammation had long been suggested as a key pathological mechanism involved in SAH pathogenesis,^[^ ^26^ ^]^ and our results revealed the differences of infiltrated immune cells between the sham and SAH group, we wondered if the dynamic remodeling of immune microenvironment played an important role in mLVs impairment. Visualization of mLECs distribution prediction showed that mLECs damage was severest at 24 h after injury (**Figure** **3**A). Therefore, we first investigated cell‐to‐cell communication among all cell types to visualize the overall situation. Through manifold learning and quantitative contrasts, CellChat identified differentially over‐expressed ligands and receptors between each cell cluster. In total, 4239 significant L‐R pairs were detected, which were further categorized into 2258 signaling pathways. Significant interactions were identified on the basis of a statistical test that randomly permuted the group labels of cells and then recalculated interaction probability.

Next, we set out to find the relationship between mLECs and other cell types. Several signals that contributed most to the outgoing or incoming signaling patterns of mLECs were identified (Figure 3B). According to the findings above, mLVs injury was most severe at 24 h after injury and monocytes were the dominant infiltrated cells at this time course, indicating that monocytes and mLECs interaction might play crucial roles in mLVs dysfunction. Therefore, by focusing on the communication between mLECs and monocytes, we found that THBS signaling pathways were especially actively involved in all signaling patterns. We further explored the potential L‐R pairs based on THBS signaling working on mLECs and calculated the contribution of these L‐R pairs. Among them, the L‐R pair “THBS1‐CD47” exhibited the strongest interaction in cell‐to‐cell communication (Figure 3C and Figure S2A,B, Supporting Information). To further assess the spatial distribution of the THBS1‐CD47 L‐R pair, we used stLearn,^[^ ^27^ ^]^ a technology especially used to analyze spatial communication, which could combine the spatial information of cells with all the predicted communication to discover the regional specificity of L‐R pairs. L‐R co‐expression enrichment among neighbor spots and cell‐type density enrichment were integrated to find hotspots within a tissue, where interactions between cell types were most likely to occur. The result showed that the THBS1‐CD47 L‐R pair significantly activated at 24 h after SAH (Figure 3D).

### 2.4 THBS1 Impaired Meningeal Lymphatic Drainage and Exacerbated Neurological Dysfunction

After discovering that the THBS1‐CD47 L‐R pair was potentially associated with mLVs injury, we then would like to assess the impact of THBS1 after SAH. We checked endogenous THBS1 alteration after SAH by using our previous mass spectrometry proteomics data from ProteomeXchange Consortium (PXD030593). Differentially expressed proteins in human CSF of SAH patients measured by mass spectrometry indicated significant up‐regulation of THBS1 protein (we used CSF samples from normal pressure hydrocephalus (NPH) patients as the control group, Figure 3E). Given that the human THBS family had three subtypes, including THBS1, THBS2, and THBS4,^[^ ^28^ ^]^ we used an ELISA kit to again test the expression of all THBS subtypes in CSF samples of SAH patients to verify if THBS1 was the key THBS member. As we expected, although THBS2 and THBS4 levels also elevated, expression of THBS1 increased most after SAH compared to the NPH group (Figure 3F and Table S1, Supporting Information). Also, higher THBS1 level was strongly connected with poorer clinical outcome in SAH patients (Figure 6F).

**Figure 4:**
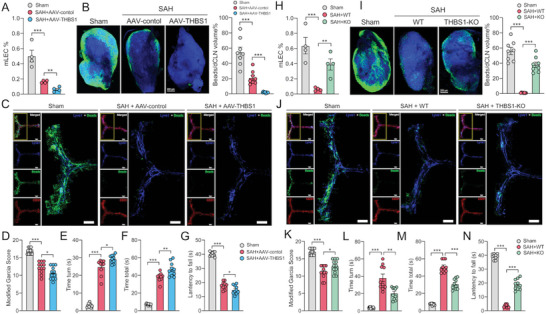
Validation of THBS1 impacting mLVs function after SAH. SAH modeling and behavioral tests were performed 4 weeks after the AAV injection. A) Flow cytometric analysis of mLECs percentage in Sham, AAV‐control, and AAV‐THBS1 group at 72 h post‐SAH, *n* = 4 per group, **p < 0.01, ***p < 0.001 by paired two‐tailed Student's *t*‐test. B) Representative images and quantification of beads accumulation in dCLNs of Sham, AAV‐control, and AAV‐THBS1 group at 72 h post‐SAH, each data point represents an average of the 2 dCLNs from one individual mouse, *n* = 8 per group, **p < 0.01, ***p < 0.001 by paired two‐tailed Student's *t*‐test. Scale bar: 200 µm. C) Representative confocal images of mLVs region of Sham, AAV‐control, and AAV‐THBS1 group at 72 h post‐SAH. An enlarged view of TS and SSS is shown on the right in each group. Lyve1 (blue), Beads (green), and CD31 (red). Scale bar: 1000 µm. D) Modified Garcia test, E) time turn, F) time total, and G) wire hanging test at 72 h after SAH revealed AAV‐THBS1 delivery aggravated short‐term neurological function compared with Sham or AAV‐control group, *n* = 10–12 per group. *p < 0.05; **p < 0.01, ***p < 0.001 by paired two‐tailed Student's *t*‐test. H) Flow cytometric analysis of mLECs percentage in Sham, SAH‐WT, and THBS1‐KO group at 24 h post‐SAH, *n* = 4 per group, **p < 0.01, ***p < 0.001 by paired two‐tailed Student's *t*‐test. I) Representative images and quantification of beads accumulation in dCLNs in sham, SAH‐WT, and THBS‐KO group at 24 h post‐SAH, each data point represents an average of the 2 dCLNs from one individual mouse. *n* = 8 per group, **p < 0.01 by paired two‐tailed Student's *t*‐test. Scale bar: 200 µm. J) Representative confocal images of mLVs region of Sham, SAH‐WT, and THBS1‐KO group at 24 h post‐SAH. An enlarged view of TS and SSS is shown on the right in each group. Lyve1 (blue), Beads (green), and CD31 (red). Scale bar: 1000 µm. K) Modified Garcia test, L) time turn, M) time total, and N) wire hanging test at 24 h after SAH revealed THBS1 knockout improved short‐term neurological function compared with Sham or WT‐SAH group. *n* = 10–12 per group. *p < 0.05; **p < 0.01, ***p < 0.001 by paired two‐tailed Student's *t*‐test.

**Figure 5:**
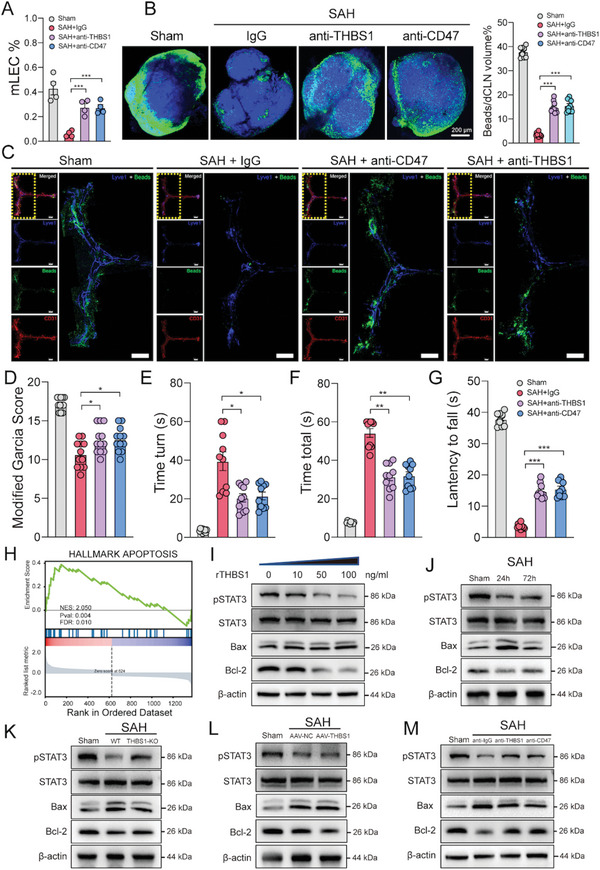
Disturbing THBS1‐CD47 interaction promoted mLVs restoration via inhibiting STAT3/BCL2‐mediated apoptosis in mLECs. A) Flow cytometric analysis of mLECs percentage in sham, SAH + igG, SAH + anti‐CD47, and SAH + anti‐THBS1 group at 24 h post‐SAH, *n* = 4 per group, ***p < 0.001 by paired two‐tailed Student's *t*‐test. B) Representative images and quantification of beads accumulation in dCLNs of Sham, SAH + igG, SAH + anti‐CD47, and SAH + anti‐THBS1 group at 24 h post‐SAH, each data point represents an average of the 2 dCLNs from one individual mouse, *n* = 8 per group, ***p < 0.001 by paired two‐tailed Student's *t*‐test. Scale bar: 200 µm. C) Representative confocal images of mLVs region of Sham, SAH + igG, SAH + anti‐CD47, and SAH + anti‐THBS1 group at 24 h post‐SAH. An enlarged view of TS and SSS is shown on the right in each group. Lyve1 (blue), Beads (green), and CD31 (red). Scale bar: 1000 µm. D) Modified Garcia test, E) time turn, F) time total, and G) wire hanging test at 24 h after SAH revealed anti‐CD47 and anti‐THBS1 therapy improved short‐term neurological function compared with Sham or SAH + igG group. *n* = 10–12 per group. *p < 0.05; **p < 0.01, ***p < 0.001 by paired two‐tailed Student's *t*‐test. H) GSEA showed apoptosis pathway was activated in mLECs after SAH. I) Representative immunoblot images showing effects of rTHBS1 (100 ng mL^−1^) treatment on pSTAT3 and Bcl‐2 inhibition in primary mLECs. J–M) Representative immunoblot images of STAT3, pSTAT3, Bax, and Bcl‐2 in different groups.

We then performed in vivo experiments to explore the role of THBS1 in mLVs injury after SAH. First, low‐dosage mouse recombinant THBS1 protein was delivered into mice cisterna magna to observe mLVs function changes. Compared to negative control (NC) mice, recombinant THBS1 (rTHBS1) protein treatment caused less beads aggregation in mLVs and dCLNs (Figure 3G,I and Figure S3A, Supporting Information) and decreased mLECs number of mLVs (Figure 3H). Second, we constructed THBS1‐overexpressing mice through the delivery of AAV‐THBS1 into mice cisterna magna, along with cultivating THBS1‐KO mice for further research. Compared to AAV‐control, mLVs injury was even worse with AAV‐THBS1 treatment after SAH (**Figure** **4**A–C and Figure S3B, Supporting Information). Conversely, THBS1‐KO partly reversed mLVs damage caused by SAH (Figure 4H–J and Figure S3C, Supporting Information). Also, neurological function tests presented similar results. Overexpression of THBS1 aggravated neurological deficits caused by SAH, while THBS1 knockout improved neurofunction (Figure 4D–G,K–N). What is more, consistent with the results of THBS1 knockout, mLVs and neurofunction impairment was reversed as well after blockage of THBS1 with THBS1‐antibody (**Figure** **5**A–G and Figure S3D, Supporting Information). Collectively, all these results proved that up‐regulated THBS1 caused mLVs structure and drainage function damage after SAH, thereby exacerbating neurobehavioral dysfunction.

### 2.5 Disturbing THBS1‐CD47 Interaction Promoted mLVs Restoration via Inhibiting STAT3/BCL2‐Mediated Apoptosis in mLECs

Previous studies reported that THBS1 could inhibit endothelial cell proliferation and chemotaxis by engaging its receptors CD47.^[^ ^29^ ^]^ Similarly, our results predicted that the THBS1‐CD47 L‐R pair played an important role in mLVs impairment and proved the injurious effect of THBS1 after SAH. We therefore moved on to discover CD47 function. Blockage of CD47 with antibody attenuated meningeal lymphatic dysfunction compared to igG treatment at 24 h post‐SAH (Figure 5A–C and Figure S3D, Supporting Information). Accordantly, the neurological function also improved significantly (Figure 5D–G).

Next, to further investigate the underlying mechanism of THBS1‐CD47 interaction‐induced injury, GSEA analysis was performed.^[^ ^30^ ^]^ The result indicated that the apoptosis pathway in mLECs was significantly activated 24 h after injury (Figure 5H). Tunel staining indicated that apoptosis was significantly activated after SAH. Overexpression of THBS1 further promoted the apoptosis of mLVs while blockage of THBS1 showed decreased Lyve1^+^Tunel^+^ cells (Figure S5A, Supporting Information). Co‐cultivation of human monocytes and LECs in vitro confirmed that the content of THBS1 in the supernatant of the co‐culture system was significantly increased under the stimulation of hemoglobin (150 µm), indicating that monocytes are one of the sources of THBS1 secretion after SAH (Figure S5B, Supporting Information). Flow cytometric analysis revealed that the apoptosis of LECs in the co‐culture system pretreated with THBS1 neutralizing antibody is significantly reduced compared to the control group (Figure S5C,D, Supporting Information). However, the precise mechanism of mLVs injury remains unknown.

Studies had demonstrated that THBS1‐CD47 interaction induced endothelial cell apoptosis through disturbing VEGF/VEGFR2 signaling, which was essential for endothelial development.^[^ ^29^ ^]^ Also, VEGFR2 inhibition was found to promote apoptosis via suppressing STAT3/Bcl‐2 signaling in osteosarcoma.^[^ ^31^ ^]^ Therefore, we hypothesized if THBS1‐CD47 interaction could medicate mLECs apoptosis via inhibiting STAT3/Bcl‐2 signaling. First, co‐immunoprecipitation (COIP) confirmed the interaction between THBS1 and CD47 in LECs (Figure S4A, Supporting Information). Second, along with increased expression of Bax, expression of pSTAT3 and Bcl‐2 were significantly decreased after rTHBS1treatment in primary mLECs in a dose‐dependent manner (Figure 5I), indicating that  rTHBS1 treatment promoted mLECs apoptosis by repressing the activation of the STAT3/Bcl‐2 signaling pathway. Finally, in SAH mice mLVs samples, the level of pSTAT3 and Bcl‐2 peaked at 24 h and then decreased at 72 h after SAH, which was consistent with GSEA results (Figure 5J). THBS1 knockout or blockage of THBS1/CD47 could all significantly increase pSTAT3 and Bcl‐2 expression (Figure 5K,M), whereas THBS1‐overexpression presented the opposite effect (Figure 5L). Taken together, all these findings verified that the THBS1‐CD47 L‐R pair promoted mLECs apoptosis via STAT3/Bcl‐2 signaling, and inhibition of this interaction might help alleviate mLVs injury.

### 2.6 Pseudotime Analysis Revealed mLECs Alteration Trace after SAH

**Figure 6:**
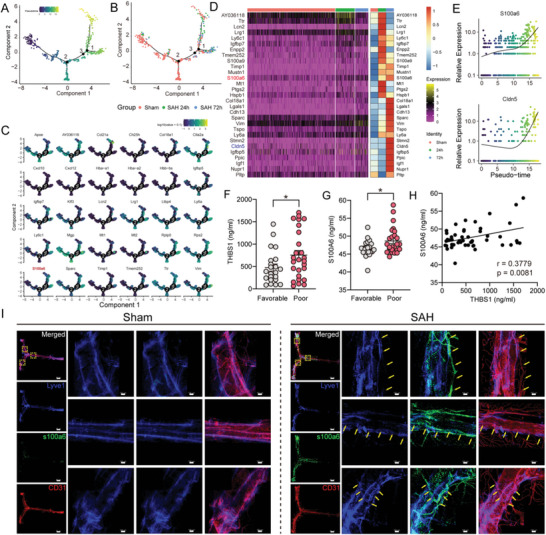
Cell trajectory analysis shows the evolution of mLECs. A) Labeling mLECs with real grouping information based on pseudotime trajectory map. B) Pseudotime trajectory map. C) Trajectory of representative genes in mLECs. D) Heatmap showing the top 30 pseudotime‐related genes. E) Representative two pseudo‐time‐related genes (S100*α*6 and Cldn5) based on *q* values. F,G) Increased expression of THBS1 and S100A6 are associated with poor prognosis, *n* = 48, *p < 0.05 by paired two‐tailed Student's *t*‐test. H) Linear relationship between THBS1 and S100A6 expression, *r* = 0.3779, *p* = 0.0081 by Spearman's rank test. H) Representative confocal images of mLVs region of sham and SAH 24 h group. Enlarged view of selected region in the merged photo (yellow dotted box) are listed on the right in each group. Lyve1 (blue), S100*α*6 (green), and CD31 (red). Yellow arrows indicate where mLVs lie. Scale bar: 800 µm for the holistic view and 100 µm for the enlarged view.

Additionally, we performed pseudotime analysis of all mLECs in different samples to reveal mLECs change trajectory during the EBI stage after SAH (**Figure** **6**A). After labeling mLECs with real grouping information, we found that the damage pattern of mLECs 24 h after SAH was consistent with the findings above. However, unexpectedly, mLECs at 72 h seemed to possess completely different cell states or characteristics compared to the sham group and 24 h group (Figure 6B). Considering that mLVs impairment was partly improved at 72 h after SAH (Figure 1I), we wondered if self‐repairing mechanisms of mLECs or other cell‐to‐cell interactions functioned at this time course, which was worth the further efforts and exploration. DEG analysis revealed the most over‐expressed genes after SAH (Figure 6C,D). Along the trajectory, the expression of S100*α*6 and Cldn5 gradually increased (Figure 6E). Previous research reported that S100A6 impaired lymphatic endothelial cells tight junction and increased the transendothelial migration of neutrophils in breast cancer,^[^ ^32^ ^]^ indicating that S100*α*6 might be a potential mLECs damage biomarker after SAH.

We then performed immunofluorescent staining of mLVs to identify S100*α*6 changes after SAH. As expected, there was a marked increase in meningeal S100*α*6 expression and more Lyve1^+^S100*α*6^+^ positive cells 24 h after injury while Lyve1^+^ cells decreased compared to the uninjured group (Figure 6I). Conversely, the expression of S100*α*6 was also significantly reduced when THBS1 was knocked out (Figure S6, Supporting Information). Also, through the ELISA assay, we confirmed increased expression of S100A6 in CSF samples of SAH patients compared to the control. Prognostic analysis suggested higher expression of S100A6 was related to a worse prognosis (Figure 6G and Table S1, Supporting Information). Meanwhile, the expression of S100A6 and THBS1 had a linear relationship (Figure 6H), which indicated that both of them might be appropriate predictors after SAH. In summary, our data illustrated that S100*α*6 might be a novel biomarker of meningeal lymphatic injury, which further supplemented the gaps in the field of mLVs annotation.

## 3 Discussion

In this study, we integrated scRNA‐seq and ST to systematically delineate the cellular, molecular, and spatial alterations of injured mLVs after SAH for the first time. Analysis of DEGs between sham and SAH groups showed that gene transcription in each cell cluster and immune microenvironment underwent widespread changes after SAH. Cell‐to‐cell communication revealed secreted protein THBS1 binding to receptor CD47 visibly activated in mLECs after SAH. We hypothesized that activation of THBS1‐CD47 interaction after SAH promoted mLECs apoptosis by inhibiting the STAT3/Bcl‐2 signaling pathway, thereby impacting mLVs function. We verified our hypothesis through both in vivo*/*vitro experiments. Our research demonstrated that multi‐omics profiling can provide more comprehensive information from different perspectives, enabling better resolution of cellular composition, decipherment of immune cell infiltration, and explanation of the underlying pathological mechanism of mLVs after SAH.

SAH is usually caused by a rupture of intracranial aneurysms. A mass of blood suddenly flushes into SAS through the ruptured aneurysm and results in subsequent brain injury.^[^ ^33^ ^]^ On one hand, massive aggregation of blood in SAS lead to rapid elevation of ICP.^[^ ^34^ ^]^ On the other hand, the breakdown of red blood cells releases several intracellular components, such as hemoglobin, into CSF, which can directly exert neurotoxic effects.^[^ ^33^ ^]^ These two processes are devastating and are the predominant causes of EBI after SAH.^[^ ^35^ ^]^ Although several endogenous mechanisms have been discovered, like erythrophagocytosis, to have the potential of clearing hemoglobin or other neurotoxicants, these mechanisms are quite easy to get overloaded or dampened.^[^ ^36^ ^]^ Therefore, it is urgent and meaningful to further discover the pathological mechanism after SAH and find out a much more effective approach for neurotoxicant removal.

The CNS is traditionally considered an immune‐privileged organ, mainly due to the lack of direct communication or connection with the peripheral immune system.^[^ ^37^ ^]^ However, the existence of lymphatic vessels (LVs) in the dura, known as mLVs, has been confirmed in both mice and humans.^[^ ^5^, ^38^ ^]^ Given that mLVs drain CSF and interstitial fluid (ISF) into dCLNs, it is reasonable to surmise that these vessels are involved in clearing waste, regulating the flow of CSF, and maintaining brain homeostasis under physiological conditions.^[^ ^5^, ^39^ ^]^ The discovery of mLVs and subsequent studies have improved our understanding of several CNS diseases including Alzheimer's disease, traumatic brain injury, and subdural hematomas.^[^ ^6^, ^40^ ^]^ As aforementioned, Chen et al. found that extravasated erythrocytes from SAS were drained into dCLNs via mLVs.^[^ ^3b^ ^]^ In addition, ablation of mLVs exacerbated EBI symptoms in mice after SAH.^[^ ^41^ ^]^ These discoveries identified mLVs as an important path for intracranial waste clearing and a promising therapeutic target for accelerating erythrocyte drainage after SAH. Nevertheless, the cellular architecture and the underlying gene regulatory features of mLVs post‐SAH have not been fully characterized. Our current project intended to apply scRNA‐Seq combined with ST to reveal the underlying mechanism of mLVs injury after SAH.

MLVs mainly lie in the dura, which is the outermost of the three layers of meninges that cover the brain and spinal cord. Unlike the adjacent brain parenchyma, a wide repertoire of immune cells is located in the dura and takes part in the constitution of meningeal immunity, which is concerned with the immune surveillance of CNS.^[^ ^42^ ^]^ Meningeal immunity has recently come under the spotlight owing to the discovery of mLVs drainage function. Immune cell population alteration, immune‐inflammatory signaling pathway activation, and reactive substance generation are activated in the brain parenchyma after SAH. Crosstalk between immune dysfunction and hyperactivation of inflammatory signals aggravates the devastating consequences of brain injury and cerebral vasospasm. Presently, research on immune inflammation after SAH mainly focuses on injury in brain parenchyma. However, the changes in the immune microenvironment in dura remain unclear. In this study, we discovered that around mLVs, neutrophils, monocytes, and macrophages were markedly infiltrated at 24 h, and pDCs and T cells gradually increased and peaked at 72 h post‐SAH. These results were consistent with the trend of immune cell infiltration in the brain parenchyma after SAH, proposing a possibility that meningeal immune cells could directly migrate to parenchyma through mLVs after SAH.

THBS1 is a member of the thrombospondin protein family.^[^ ^43^ ^]^ It is involved in tissue remodeling and with implications for tumor formation, wound healing, and embryonic development.^[^ ^44^ ^]^ THBS1 exerts different effects by binding to different cellular receptors.^[^ ^45^ ^]^ Previous studies have shown that THBS1 is highly expressed in lymphoma,^[^ ^46^ ^]^ breast cancer,^[^ ^47^ ^]^ and oral squamous carcinoma,^[^ ^48^ ^]^ promoting tumor cell adhesion, proliferation, apoptosis, invasion, and metastasis. We discovered that THBS1 expression was elevated in CSF after SAH, and its level was associated with the prognosis of SAH patients, indicating that THBS1 might be a potential biomarker of SAH. Also, THBS1 is the first identified endogenous inhibitor of angiogenesis.^[^ ^29a^ ^]^ It plays only a minor role in developmental angiogenesis, but it is an important regulator of pathological angiogenesis in the adult. Through interacting with its receptors CD36 and CD47, THBS1 can block endothelial cell proliferation and chemotaxis stimulated by fibroblast growth factor‐2 and VEGF and further induce endothelial cell apoptosis.^[^ ^29a^ ^]^ By combining scRNA‐Seq and ST analysis, our present research predicted that mLECs apoptosis induced by THBS1‐CD47 interaction might be the key mechanism of mLVs dysfunction after SAH. After several in vivo*/*vitro experiments, we proved this hypothesis and further discovered that the downstream STAT3/Bcl‐2 signaling pathway participated in mLECs apoptosis.

Up to now, there is no research on mLVs injury after SAH. Our research attempted to use a multi‐omics study to describe the atlas of mLVs after SAH, to find potential injury mechanisms, and to mine more biological markers. S100*α*6 protein belongs to A group of the S100 protein family of Ca^2+^ binding proteins.^[^ ^49^ ^]^ As an intracellular protein, S100*α*6 has been implicated in the regulation of several cellular functions, such as proliferation, apoptosis, cytoskeleton dynamics, and cellular response, to different stress factors.^[^ ^50^ ^]^ Previous studies revealed S100*α*6 impaired lymphatic endothelial cells tight junction and increased trans‐endothelial migration of neutrophils in breast cancer.^[^ ^32^ ^]^ Meanwhile, the dosage of serum S100*α*6 might aid in diagnosis in oncology.^[^ ^50b^ ^]^ Our study identified increased expression of S100*α*6 both in mLVs and human CSF after SAH and its correlation with poor prognosis, indicating S100*α*6 might be a valuable biomarker.

There are several limitations to our study. First, our study provided strong data to support an association among SAH, mLVs, and THBS1/CD47 L‐R pair because THBS1 or CD47 blockage improves mLVs in SAH mice. However, if these beneficial effects are directly mediated by mLECs THBS1/CD47 signal is not known. Since THBS1/CD47 L‐R pair affects many cell types, it is also possible that changes in mLVs and mLECs are indirectly influenced by THBS1/CD47 in other cell types. Second, the possible sources of THBS1 or S100A6 after SAH should be discussed.

In conclusion, for the first time, we illustrated a landscape of spatial and cellular alterations of mLVs at an early stage after SAH. We discovered significant mLECs population change with the remodeling of the surrounding immune microenvironment. Meanwhile, overexpression of THBS1 and interaction between THBS1‐CD47 led to mLECs apoptosis possibly through STAT3/Bcl‐2 signaling. Taken together, our findings may provide new perspectives for the following SAH‐related research and treatment strategies for SAH intervention based on the improvement of impaired mLVs.

## 4 Experimental Section

### Clinical Sample Collection and Patient Information

Patients with aneurysmal SAH were diagnosed by computed tomography (CT) and digital subtraction angiograph (DSA) performed within 24 h after admissions. The inclusion and exclusion criteria were consistent with the previous study.^[^ ^51^ ^]^ All CSF samples of SAH patients were collected before coiling or clipping through a lumbar puncture or lumbar drainage (LD) or external ventricular drainage (EVD) within 48 h after SAH. All CSF sample collection and patient information were consistent with the previous report.^[^ ^3a^ ^]^

### Mass Spectrometry of Human CSF

The previous mass spectrometry proteomics data from ProteomeXchange Consortium (PXD030593) was used for downstream analysis. Used the hiplot tool to present relevant gene expression in the form of a volcano plot.^[^ ^52^ ^]^

### ELISA Assay

The concentrations of human CSF THBS1 (EK0899, Boster Biotech, Wuhan), THBS2 (EK0642, Boster Biotech, Wuhan), THBS4 (CSB‐EL023490HU, Cusabio Biotech, Wuhan), and S100A6 (CSB‐EL020634RA, Cusabio Biotech, Wuhan) were determined using ELISA kits. Human CSF samples (100 µL) were used for the analysis. All procedures were performed according to the manufacturer's instructions.

### Animals

Specific pathogen‐free, C57BL/6 male mice (6–8 weeks old) were purchased from Shanghai SLAC Laboratory Animal Co. Ltd. For AAV injection, C57BL/6 mice were injected with AAV‐Ctrl, AAV‐THBS1 at 6 weeks of age, when the weight was ≈20 g. SAH modeling and behavioral tests were performed 4 weeks after the AAV injection. C57BL/6 mice were injected into with recombinant mice THBS1 protein or vehicle (PBS) via cisterna magna 1 day in advance before the sacrifice. THBS1^−/‐^ mice were established on a C57BL/6 background and purchased from Gempharmatech Co., Ltd (#KO‐T014586, NCBI:21 825, MGI:98 737). Mice were housed in the animal facility with controlled habituation and temperature, on 12‐h light versus dark cycles, and fed with regular rodents' chow and sterilized tap water ad libitum. Mice were allowed to accommodate for 2 weeks before experimental procedures.

### Induction of SAH

The prechiasmatic SAH mice model was created as previously described.^[^ ^53^ ^]^ In brief, after induction of anesthesia with 1% pentobarbital, the head was fixed in a stereotactic apparatus (RWD Life Science Co., Ltd). With a midline incision, the skin overlying the anterior skull was opened. A burr hole was drilled into the skull 4.5 mm anterior to the bregma with a caudal angel of 40° using a 0.9‐mm drill. Blood (60 µL) was withdrawn from a C57BL/6 WT blood donor and injected over a 10‐s period with a 26‐gauge needle advanced through the burr hole at a 40° angle until reaching the base of the skull. For antibody injection, a mixture of 10 µg mL^−1^ CD47 antibody (Invitrogen, 16‐0479‐85) or THBS1 antibody (Genetex, GTX21823) or IgG (Invitrogen, 14‐4714‐85) with 60 µL autologous blood was injected into the prechiasmatic cisternae. The needle was left in place for 5 min before retraction to avoid backflow. Mice that underwent the same procedure, without blood and antibody/IgG injections, served as the sham group.

### Intra‐Cisterna Magna Injections

Mice were anesthetized by intraperitoneal (i.p.) injection with 1% pentobarbital. The skin of the neck was shaved and cleaned with iodine and 70% ethanol, and an ophthalmic solution (erythromycin ointment) was placed on the eyes to prevent drying. Stereotaxic frames were used to secure the mouse's head, and a midline incision was made in the skin. The muscle layers were retracted and the cisterna magna exposed. Using a Hamilton syringe (coupled to a 33‐gauge needle), the volume of the desired solution was injected into the CSF‐filled cisterna magna compartment. For THBS1 or Control AAV delivery experiments, 2 µL of artificial CSF containing 10^13^ genome copies per mL of AAV9‐THBS1, or AAV‐Con (AAV9, adeno‐associated virus serotype 9; purchased from PackGene Biotech, Guangzhou), were injected into the cisterna magna CSF at a rate of 2 µL min^−1^. For recombinant protein experiments, recombinant mice THBS1 protein (150 µg kg^−1^, R&D Systems, 7859‐TH‐050, USA) or vehicle (PBS) were injected into cisterna magna CSF at a rate of 2 µL min^−1^. For the beads experiments, 2 µL of FluoSpheres carboxylate 0.5 µm‐beads 505/515 (Invitrogen, F8813) were injected at a rate of 2 µL min^−1^. The needle was inserted into the cisterna magna through the retracted muscle in order to prevent backflow upon needle removal. The neck skin was then sutured and allowed to recover on a heating pad until fully awake.

### Neurological Scores and Behavioral Testing

Neurological scores and all behavioral assessments of each group were carried out at similar and relatively fixed time during daylight hours in a blinded fashion.

### Neurological Scores

The Modified Garcia (ranging from 0 to 18) was conducted to evaluate short‐term neurological function. The scoring system was comprised of six sub‐tests including response capacity, alertness, coordination, motor skills, complex movements, and coordination.^[^ ^54^ ^]^ A blinded investigator performed the neurological examination, which was scored 24 and 72 h after SAH. A higher score represented a better neurological function.

### Pole Test

A pole test was performed 24 and 72 h postoperatively. Animals are placed on top of a 50‐ to 55‐cm vertical pole with a diameter of 8 to 10 mm and trained to turn around and descend the pole. The scoring started when the animal initiated the turning movement. The time to make a complete 180° turn (i.e., *T*_turn_) and latency to reach the ground (i.e., *T*_total_) were recorded. If the animal cannot turn but instead descends with a lateral body position, then *T*_total_ was usually attributed to *T*_turn_. When an animal makes a turn, descends halfway, and falls, the recorded time stops when the animal reaches the floor. The maximum time to perform the test was set to 60 s. Mice were trained three times daily for 3 days before the start of testing.

### Wire Hanging Test

Wire Hanging is a simple test that evaluates grip strength, balance, and endurance. Mice were trained to suspend their body by holding on to a single wire stretched between two posts 50 to 60 cm above the ground. To prevent the animal from using all four paws, the hind limbs were gently covered with adhesive tape. Between the two posts was a pillow to avoid injury when the mice fell. “Latency to fall” was the primary end point used to assess motor performance. The maximum time to perform the test was set to 60 s. Mice were trained three times daily for 3 days before the start of testing.

### Tissue Collection

Mice were euthanized with isoflurane and dCLNs were dissected under the microscope. Subsequently drop‐fixed in 4% paraformaldehyde (PFA) for 2 h at 4 °C and then the CUBIC clearance protocol was performed as described below in the dCLN clearance methods sections.^[^ ^55^ ^]^ For meningeal whole‐mount collection, mice were transcardially perfused with 20 mL cold 1× PBS, then skin and muscle were stripped from the outer skull, and the skullcap was removed with surgical scissors and fixed in 2% PFA for 12 h at 4 °C. Then the meninges (dura mater and arachnoid mater) were carefully dissected from the skullcaps with Dumont #5 forceps (Fine Science Tools). Meningeal whole‐mounts were then moved to PBS and 0.05% azide at 4 °C until further use.

### dCLN Clearance

Consistent with the previous report,^[^ ^41^ ^]^ dCLN clearance was performed following the published CUBIC protocol with modifications.^[^ ^55^ ^]^ In brief, nodes were incubated in 50% reagent 1 (prepared 1:1 with dH2O) for 1 day at 37 °C, shaking, with DAPI (1:1000). Nodes were then transferred to reagent 1 for 1 day at 37 °C, shaking, with DAPI (1:1000). Nodes were washed two times in PBS + 0.01% sodium azide for 2 h and overnight with DAPI (1:1000) at 37 °C. Then nodes were incubated with 50% reagent 2 (prepared 1:1 with dH2O) for 1 day at 37 °C with DAPI (1:1000). Finally, nodes were incubated with reagent 2 for 1 day at 37 °C. Nodes were placed in eight well chambers (155 411, Thermo Fisher) with mineral oil and imaged with confocal microscopy.

### Immunofluorescence, Imaging, and Quantification

For immunofluorescence staining, meningeal whole‐mounts in PBS and 0.05% azide were blocked with either 2% donkey serum or 2% goat serum, 1% bovine serum albumin, 0.1% triton, 0.05% tween‐20, and 0.05% sodium azide in PBS for 1.5 h at room temperature. This blocking step was followed by incubation with appropriate dilutions of primary antibodies: Rat anti‐Lyve1(Santa Cruze, sc‐65647, 1:200), Goat anti‐CD31 (R&D Systems, AF3628, 1:100), and Rabbit anti‐S100*α*6 (Abcam, ab181975, 1:200) in the same solution used for blocking overnight at 4 °C. Meningeal whole‐mounts or brain tissue sections were then washed three times for 10 min at room temperature in PBS and 0.05% tween‐20, followed by incubation with the appropriate donkey Alexa Fluor 405 or 488 or 594 anti‐rat, ‐goat,‐rabbit (Thermo Fisher Scientific, 1:1000) for 2 h at RT in the same solution used for blocking. For Tunel staining, apoptotic cells were detected according to the manufacturer's instructions using the apoptotic kit (Beyotime Biotech, C1090, China). The whole mounts were then washed three times for 10 min at RT in PBS. The tissue was then transferred to PBS and mounted with ProLong Gold antifade reagent (Invitrogen, P36934) on glass slides with coverslips. Slide preparations were stored at 4 °C and imaged using a Lecia DMI8 confocal microscope and LAS AF software (Leica Microsystems) within 1 week of staining. Quantitative analysis of the acquired images was performed using Fiji software. For lymph nodes, the percent volume of microbead coverage in cleared dCLN was assessed by creating a 3D reconstruction of the node and then calculating the volume covered by beads divided by the total volume of the node using Fiji. The right and the left dCLN percent volume were averaged together for each mouse. For assessment of mLV coverage and complexity, images of the meningeal whole‐mounts were acquired using a confocal microscope and Fiji was used for quantifications. The entire meningeal whole‐mount was traced and used for quantification. The percent area coverage of Lyve1 was used to determine the coverage of the lymphatic vessels. When applicable, the same images were used to assess the percentage of field coverage by Lyve1^−^CD31^+^ vessels. All meningeal whole mounts used for the quantification of lymphatic morphology were imaged with identical confocal settings and Fiji parameters.

### Flow Cytometric

Mice were euthanized by Euthasol injection (i.p.) and transcardially perfused with 20 mL cold 1× PBS for 5 min. Heads were removed and skulls were quickly stripped. Mandibles were removed, as well as all skull material rostral to maxillae. Surgical scissors were used to remove the top of the skull, cutting clockwise, beginning and ending inferior to the right post‐tympanic hook. Meninges (dura mater, arachnoid, and pia mater) were carefully removed from the interior aspect of the skulls and surfaces of the brain with Dumont #5 forceps (Fine Science Tools). Meninges were gently pressed through 70‐µm nylon mesh cell strainers with a sterile plastic plunger (BD Biosciences) to yield a single‐cell suspension. For lymphatic endothelial cell isolation, meninges (along with diaphragm and ear skin) were digested for 30 min in 0.60 U mL^−1^ of Liberase TM (Roche) and 60 U mL^−1^ of DNase 1. Cells were then centrifuged at 300 RCF at 4 °C for 10 min, the supernatant was removed and cells were resuspended in ice‐cold FACS buffer (pH 7,4; 0.1 m PBS; 1 mm EDTA; 1% BSA). Cells were stained for extracellular marker with antibodies to anti‐CD45‐PE‐Cyanine5 (1:200, clone 30‐F11, eBiosciences), anti‐CD31‐Alexa Fluor 647 (1:200, clone 390, BD Biosciences), and anti‐LYVE1‐ Alexa Fluor 488 (1:200, clone ALY7, eBioscience). Experiments were performed on meninges from *n* = 2 mice in the sham group and *n* = 1 mice in the SAH group. For cell apoptosis analysis, the human lymphatic endothelial were plated in a 6‐well plate with 10^6^ cells. After 24 h, the cells were treated with normal medium or conditioned medium (CM) with or without THBS1 antibody (5 µg mL^−1^, Genetex, GTX21823) for another 48 h. Cells were collected using trypsin–EDTA and resuspended in annexin buffer and incubated for 15 min with Annexin V (BD Biosciences) or PI (eBioscience) according to the manufacturer's protocol. The apoptotic cells were analyzed using a FACScan flow cytometer (BD Biosciences). FACS analyses were reproduced by three independent experiments. Data processing was done with Excel and statistical analysis was performed using GraphPad Prism.

### Primary Meningeal Lymphatic Endothelial Cells Culture

For mLEC culture, meninges were dissected from P0‐P1 C57/BL6 mice. To obtain a suspension of meningeal LECs, skull caps were quickly collected, and meninges (dura mater and arachnoid) were dissected using Dumont no. 5 and no. 7 fine forceps in a complete medium composed of Dulbecco's modified Eagle's medium (DMEM) (Gibco) with 2% FBS, 1% l‐glutamine (Gibco), 1% penicillin/streptomycin (Gibco), 1% sodium pyruvate (Gibco), 1% non‐essential amino‐acids (Gibco), and 1.5% Hepes buffer (Gibco). Meninges were then incubated with 1 mL DMEM with 0.60 U mL^−1^ of Liberase TM (Roche) and 60 U mL^−1^ of DNase 1 for 30 min at 37 °C. Cell suspensions were then pooled into a single tube after filtration through a 70‐µm nylon mesh cell strainer. Cells were then centrifuged at 300 RCF at 4 °C for 10 min, the supernatant was removed and cells were resuspended with 1 mL ice‐cold magnetic separation buffer. Added 5 µL rat anti‐lyve1(Santa Cruze, sc‐65647, 1:200) antibody to the cell suspension. Vortexed briefly to mix and incubate at 4 °C for 15 min. Washed cells by adding 10 mL of magnetic separation buffer to the tube and centrifuged at 600 RCF for 5 min at 4 °C. Carefully aspirated the supernatant and resuspend the cells in 1 mL of magnetic separation buffer. Added 100 µL of anti‐rat IgG beads (Miltenyi Biotec, cat.no 130‐048‐502) to the cells, vortexed briefly to mix, and incubated for 15 min at 4 °C. Added 10 mL of magnetic separation buffer to the cells and centrifuged at 4 °C, 600 RCF for 5 min. According to the manufacturer's instructions, MACS sorting was carried out by using the magnetic beads matched with the anti‐rat IgG beads. Lyve1‐positive cells were collected and resuspended in mLEC culture medium, inoculated in a culture dish, and the medium was changed every 2 days.

### THP1 Conditioned Medium Preparation

Human monocyte THP1 cells were cultured in RPMI 1640 medium containing 10% FBS (VivaCell, Shanghai, China) and 1% penicillin‐streptomycin. For CM collection, THP1 cells were plated in a 6‐well plate with FreeStyle expression medium (12 338 026; Gibco, Thermo Fisher Scientific, USA) for 24 h. The CM were collected 24 h later, centrifuged at 15 000 × *g* for 5 min, and filtered through a 0.22‐mm filter. Human lymphatic endothelial cells were exposed to fresh medium mixed with CM at a ratio of 1:1 (v/v) for the following process.

### Co‐Immunoprecipitation

10^6^ human lymphatic endothelial cells were lysed using 400 µL of lysis buffer (Tris‐HCL HCl 50 mm, pPH 7.4, Nacl NaCl 150 mm, sodium deoxycholate 0.25%, NP‐40 1%, EDTA 1 mm, PMSF 1 mm, Aprotinin 1 mg mL^−1^, leupeptin 1 mg mL^−1^, pepstatin 1 mg mL^−1^) on ice for 30 min and centrifuged at 16 113.6 rcf for 15 min. The supernatant was incubated with CD47 (Invitrogen, MA5‐11895) or IgG (Abclone, AC005) antibodies overnight and followed by protein A/G plus agarose bead (Santa Cruz Biotech., sc‐2003) for another 4 h. After washing three times with ice‐cold PBS, parvalbumin (1 ug mL^−1^) was added for 2 h. Agarose beads were washed three times with PBS and followed by SDS‐PAGE and immunoblot analysis with indicated antibodies.

### Western Blot Analysis

Western blot was used to analyze the lymphoid apoptosis pathway‐associated protein changes in meninges at different time points after SAH/sham surgery. Briefly, after euthanasia and perfusion with cold PBS, the mouse meninges were harvested, and total protein was extracted with RIPA lysis buffer and 1% PMSF. The protein was separated by 10% SDS‐PAGE at 120 V for 90 min, and the separated proteins were transferred to PVDF membranes. Then, the PVDF membranes were blocked and incubated with primary antibodies (rabbit anti‐STAT3 (1:1000, Abclone, cat no. A1192, China); rabbit anti‐ Phospho‐STAT3‐Y705 (1:1000, Abclone, cat no.AP0070, China); rabbit anti‐Bcl2 (1:1000, Abcam, cat no. ab182858); rabbit anti‐Bax, (1:1000, Abcam, cat no. ab182734); and rabbit anti‐*β* actin pAb (1:1000, diagbio, cat no. db10001, China)) on a shaker overnight at 4 °C. After being sufficiently washed, the membranes were incubated with horseradish peroxidase‐conjugated secondary antibodies (1:5000, beyotime, cat no. A0208) for 1 h at room temperature. Experiments were performed on meninges from *n* = 3 mice per group. A ChemiDoc imaging system (Bio‐Rad) was used to visualize the membranes, and ImageJ software was used to calculate the grey values of the bands.

### Single‐Cell RNA Sequencing

Freshly prepared meningeal cell suspensions were performed immediately according to the manufacturer's protocol of 10× Chromium 3' v3 kit (10× Genomics, Pleasanton, CA). Libraries were sequenced on the Illumina NovaSeq 6000 platform at Novogene, Beijing, China. On average, each sample generated about 709 m reads (SD = 121 m).

### Read Alignment, Quality Control, and Clustering Analysis

The raw gene expression matrix was generated via CellRanger. Doublets were removed through Scrublet.^[^ ^56^ ^]^ Then filtered counts matrix was converted to Seurat object using the Seurat R package (version 4.0.1). The cells with gene numbers >7000, or <200, or >20% mitochondrial counts were filtered. Each sample data was normalized via LogNormalize method and the top 2000 highly variable genes were selected for subsequent dimensional reduction. The batch effect across samples was removed via the integration method implemented in the Seurat package. PCA (*n* = 30) and the UMAP were used to reduce the dimensionality and visualize the cell distribution. Cluster‐specific genes were identified using the Wilcox rank‐sum test implemented in the FindAllMarkers function (fold change ≥ 2 and adjusted *p* ≤0.05). Cell clusters were annotated to known cell lineages using well‐recognized marker genes.

### Cell–Cell Communication Analysis

To analyze cell–cell interactions in the microenvironment, CellChat was used to infer ligand–receptor pairs between mLECs and other cell types.^[^ ^57^ ^]^ In brief, normalized expression values of each sample were fed into CellChat, and identifyOverExpressedGenes, identifyOverExpressedInteractions, as well as projectData functions were used to conduct preprocessing. Core functions “computeCommunProb,” “computeCommunProbPathway,” and “aggregateNet” were applied to infer cell‐cell communication networks. Significant interactions were defined with *p* ≤ 0.05. Finally, “netAnalysis_signalingRole_heatmap” function was used to determine the senders and receivers in the network.

### Spatial Transcriptomics Sequencing and Data Processing

Used OCT‐embedded frozen meninge for ST detection. The capture of gene expression information for ST slides was performed by the Visium Spatial platform of 10× Genomics through the use of spatially barcoded mRNA‐binding oligonucleotides in the default protocol. Raw sequencing reads of spatial transcriptomics were quality checked and mapped to the pre‐built mm10 reference genome by Space Ranger v1.1.

### Filtering, Normalization, and Deconvolution of Visium Data

The gene‐spot matrices generated after ST data processing were analyzed with the Seurat package (version 4.0.1) in R. Spots were filtered for gene counts <500, mitochondrial counts >25%, or red cell counts >20%. Normalization across spots was performed with the SCTransform function. Dimensionality reduction and clustering were performed with PCA with the top 30 PCs. Spatial feature expression plots were generated with the SpatialFeaturePlot function in Seurat (version 4.0.1). RCTD was used to deconvolute each spot to predict the underlying composition of cell types.^[^ ^25^ ^]^

### Cell Trajectory Analysis

The Monocle2 package was used for pseudotime trajectory construction.^[^ ^58^ ^]^ Briefly, the differentialGeneTest function was used to identify DEGs across cell types. The top 1000 genes based on *q* value were selected as ordering genes. DDRTree method was applied to reduce dimension. orderCells function was used to order cells with the sham group as root.

### Gene Set Enrichment Analysis

The marker genes for each group of the mLEC cluster were identified by FindMarkers from Seurat with a threshold of min.pct = 0.1 and logFC.threshold = 0 and then sorted according to avg_log2FC. The GSEA software (v4.1.0) was used to identify the Kyoto Encyclopedia of Genes and Genomes pathways.

### Statistical Analysis

Statistical analysis was performed using SPSS analysis tools (IBM Corp.) or Prism 9 software program (GraphPad Software). All data are presented as the mean ± SEM. To assess the statistical significance between the two groups, a two‐tailed unpaired Student's *t*‐test (for parametric analysis) or Mann–Whitney U test (for nonparametric analysis) was performed. One‐way analysis of variance (ANOVA) followed by Tukey's or Dunnett's multiple comparisons test were used to detect the differences in the results between groups. The correlation between the expression profiles of CSF biomarkers and the prognosis of SAH patients was analyzed using Spearman's rank test. *p* values less than 0.05 were considered to indicate statistical significance. No sample outliers were excluded. Individual in vitro experiments were performed at least three times with similar results.

### Ethics Statement

All studies concerning human information and tissues were approved by the Ethics Committee of the Second Affiliated Hospital of Zhejiang University School of Medicine (No.2020‐892) and is registered as an observational study in ClinicalTrail.gov (No. NCT04938414). All of the patients signed informed consent. All animal procedures were approved by the second affiliated hospital of Zhejiang university – Animal Ethics Committee (Approval no. AIRB‐2022‐0698) and were performed according to the Guiding Principles for the Care and Use of Laboratory Animals Approved by Animal Regulations of National Science and Technology Committee of China.

## Conflict of Interest

The authors declare no conflict of interest.

## Author Contributions

X.W., A.Z., Q.Y., and Z.W. contributed equally to this work. X.W., A.Z., JM.Z., S.C., and K.W. designed this study. J.L., S.X., JY.Z., Y.Q., J.Z., Y.F., and Y.L. supervised this study. Q.Y., JH.Z., P.X., and J.W. coordinated the sample collection. X.W., Q.Y., H.L., JH.Z., and Y.L. performed all experiments. A.Z., Q.Y., and K.W. contributed to bioinformatics and statistical analysis. S.X. and Z.W. provided technical support for scRNA‐Seq and ST data analysis. X.W., A.Z., Q.Y., and JM.Z. wrote the original manuscript. Y.F., H.L., Y.Q., J.L., K.W., and JW.Z. reviewed and polished the manuscript.

## Supporting information

Supporting Information

## Data Availability

The data that support the findings of this study are available from the corresponding author upon reasonable request.
