# Supplementary material for: Single‐Cell RNA Sequencing and Spatial Transcriptomics Reveal Pathogenesis of Meningeal Lymphatic Dysfunction after Experimental Subarachnoid Hemorrhage
Source: Adv Sci (Weinh). 2023 May 21;10(21):2301428. doi: 10.1002/advs.202301428 (PMC10375135; doi:10.1002/advs.202301428)
Supplement: Supplementary file 1 — Supporting Information [file ADVS-10-2301428-s001.pdf]

ADVANCED SCIENCE
Open Access

Supporting Information

for *Adv. Sci.*, DOI 10.1002/advs.202301428

Single-Cell RNA Sequencing and Spatial Transcriptomics Reveal Pathogenesis of Meningeal Lymphatic Dysfunction after Experimental Subarachnoid Hemorrhage

Xiaoyu Wang, Anke Zhang, Qian Yu, Zelin Wang, Junjie Wang, Penglei Xu, Yibo Liu, Jianan Lu, Jingwei Zheng, Huaming Li, Yangjian Qi, Jiahao Zhang, Yuanjian Fang, Shenbin Xu, Jingyi Zhou, Kaikai Wang, Sheng Chen* and Jianmin Zhang**

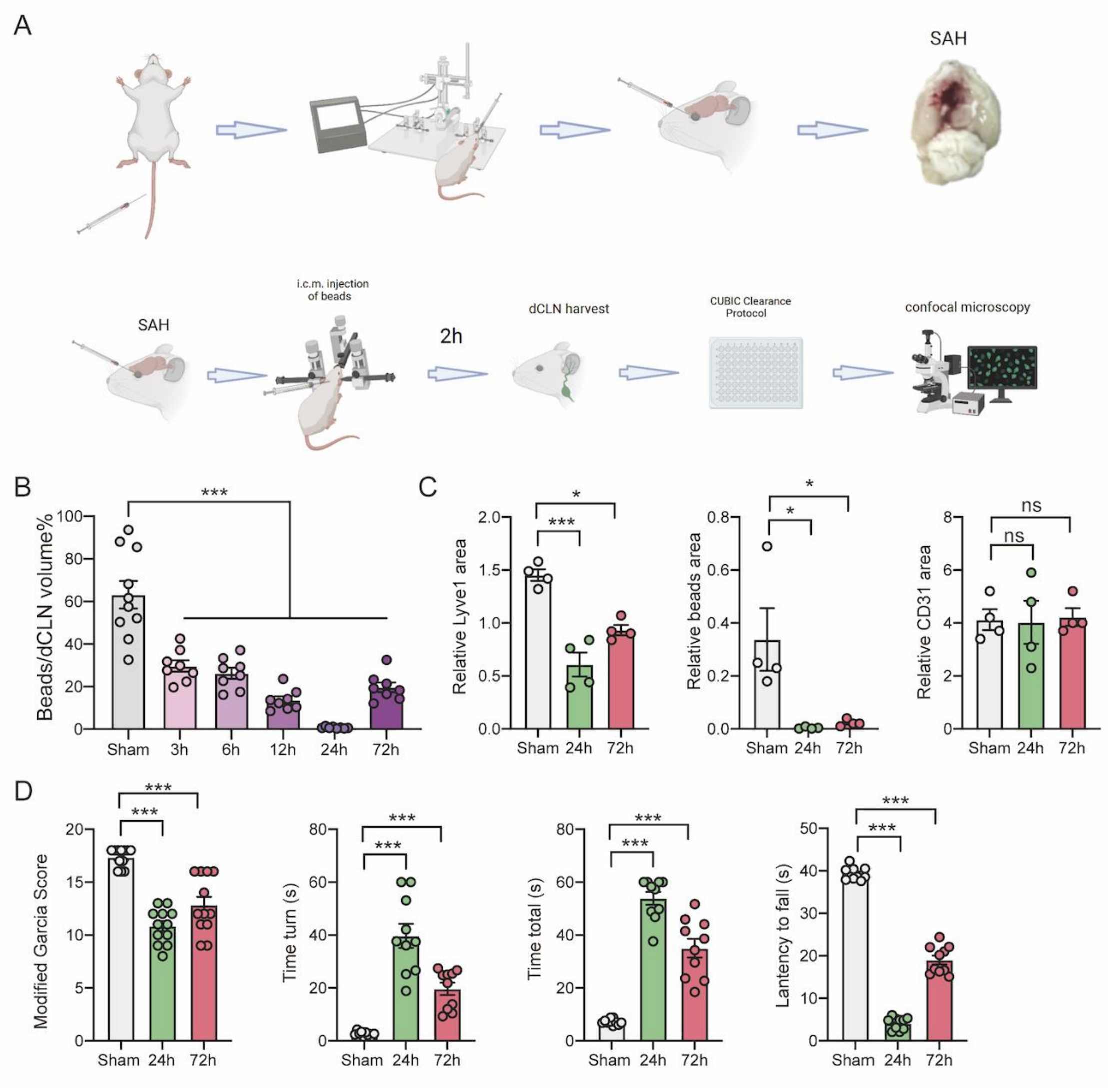

Supplementary Figure1 A) Schematic of SAH modeling and experimental layout. B) Quantification of beads accumulation in dCLNs at 3h, 6h, 12h, 24h and 72h post SAH related to Figure 1B. Each data point represents an average of the 2 dCLNs from one individual mouse (Sham n = 10, 3h n = 8, 6h n = 8, 12h n = 8, 24h n = 8, 72h n = 8), ns, not significant; *** $p < 0.001$ by one-way ANOVA and Tukey's post hoc test. C) Percent coverage area of Lyve1, Beads and CD31 fluorecence in Sham, 24h and 72h post SAH groups related to Figure 1I, n=4 per group, ns, not significant; * $p < 0.05$, *** $p < 0.001$ by paired two-tailed Student's t test. D) Modified Garcia test, Polo test and Wire hanging test results at 24h and 72h post SAH compared with Sham, n=10-12 per group, *** $p < 0.001$ by paired two-tailed Student's t test.

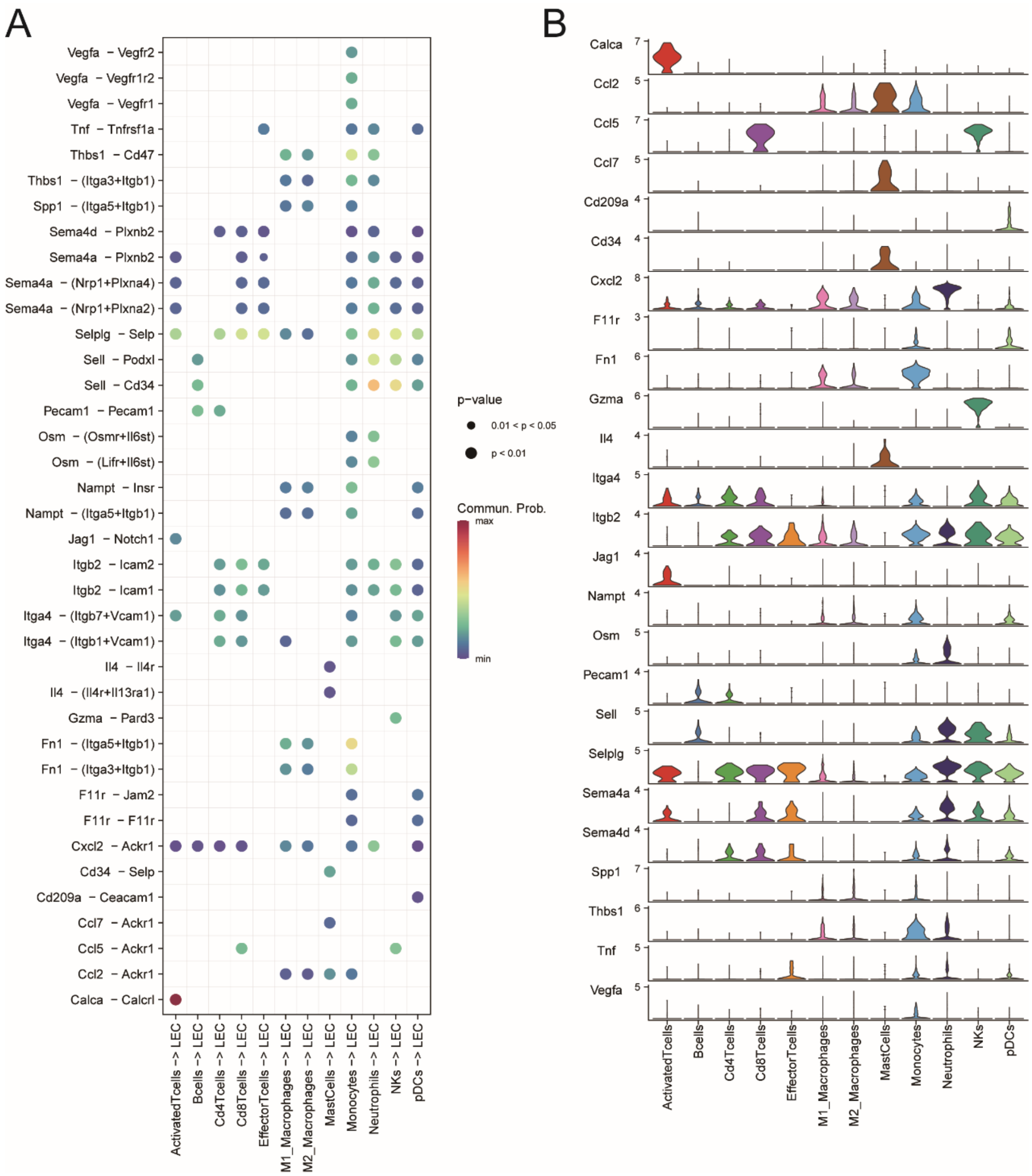

Supplementary Figure2 A) Dot plots generated by CellChat showing potential ligand-receptor pairs between mLEC and all detected cellular types in SAH 24h group. Dots are colored by communication probabilityof ligand-receptor pair between two cell types. Dots sizerepresent p value. B) Violin plots show expression of selected genes in different immune cell types.

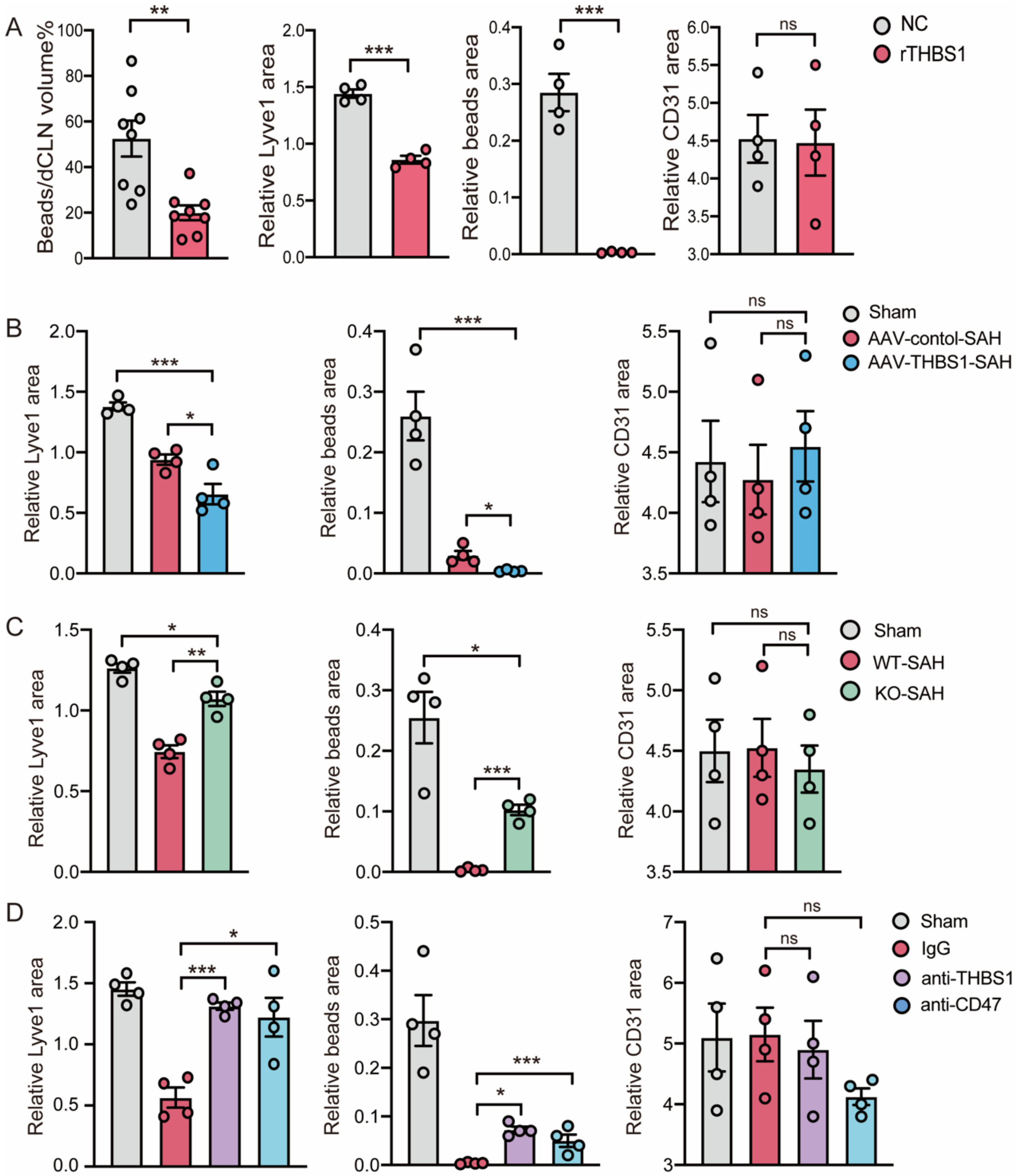

Supplementary Figure3 A) Quantification of beads accumulation in dCLNs in NC and rTHBS1 groups related to Figure 3I, n=8 per group, ** $p < 0.01$ by Student's t test; Percent coverage area of Lyve1, Beads and CD31 fluorecence in NC and rTHBS1 groups related to Figure 3G, n=4 per group, ns, not significant; *** $p < 0.001$ by paired two-tailed Student's t test. B) Percent coverage area of Lyve1, Beads and CD31 fluorecence in Sham, SAH + AAV-control and SAH +AAV-THBS1 groups related to Figure 4C, n=4 per group, ns, not significant; * $p < 0.05$, *** $p < 0.001$ by paired two-tailed Student's t test. C) Percent coverage area of Lyve1, Beads and CD31 fluorecence in Sham, SAH + WT and SAH + THBS1-KO groups related to Figure 4J, n=4 per group, ns, not significant; * $p < 0.05$, ** $p < 0.01$, *** $p < 0.001$ by paired two-tailed Student's t test. D) Percent coverage area of Lyve1, Beads

and CD31 fluorecence in Sham, SAH + igG, SAH + anti-CD47 and SAH + anti-THBS1 groups related to Figure 5C, n=4 per group, ns, not significant; * p < 0.05, *** p < 0.0001 by paired two-tailed Student's t test.

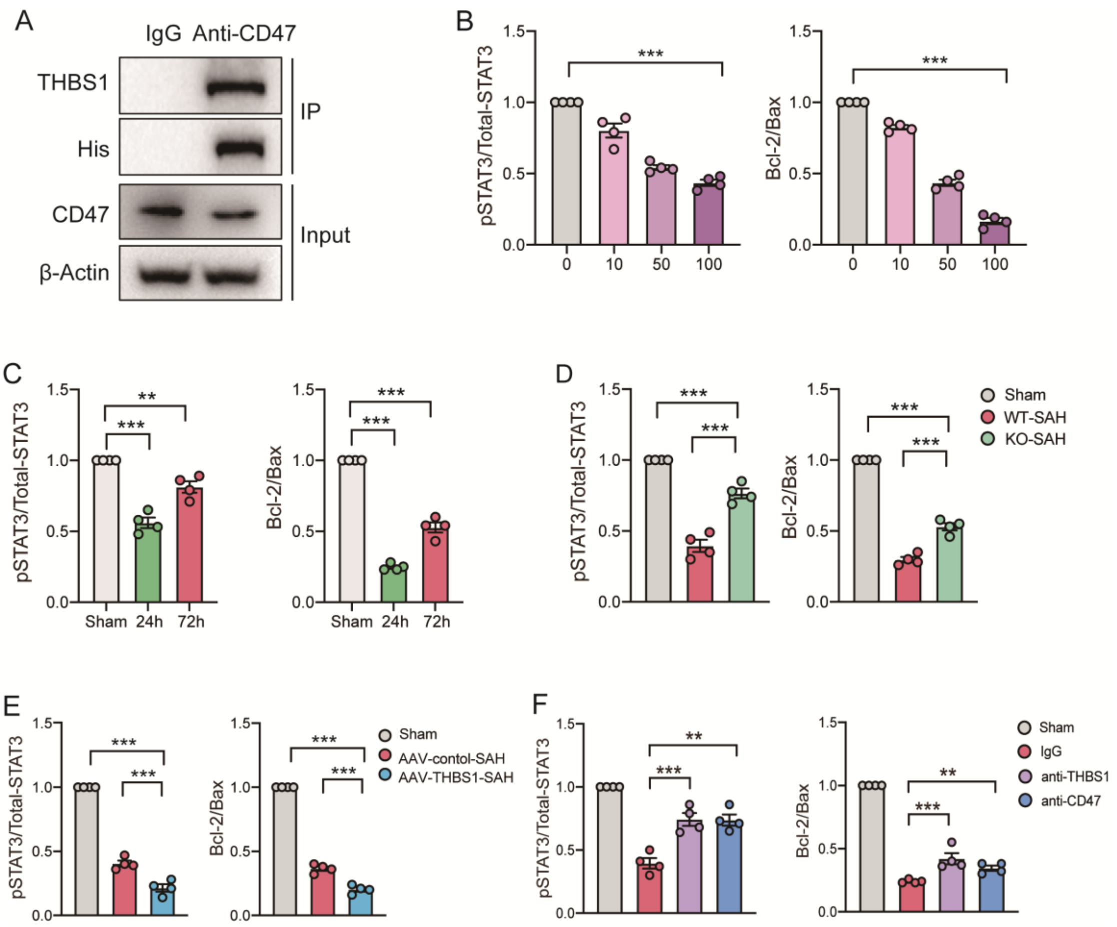

Supplementary Figure4 A) Immunoprecipitation followed by western blot analysis revealed the interaction between THBS1 and CD47 in human LECs. B) Quantification of pSTAT3/Total-STAT3 and Bcl2/Bax in primary mLECs with 0, 10, 50, 100 ng/ml rTHBS1 treatment related to Figure 5I, n=4 per group, *** p < 0.001 by paired two-tailed Student's t test. C) Quantification of pSTAT3/Total-STAT3 and Bcl2/Bax in Sham, 24h and 72h post SAH groups related to Figure 5J, n=4 per group, ** p < 0.01, *** p < 0.001 by paired two-tailed Student's t test. D) Quantification of pSTAT3/Total-STAT3 and Bcl2/Bax in Sham, SAH+WT and SAH+THBS1-KO groups related to Figure 5K, n=4 per group, *** p < 0.001 by paired two-tailed Student's t test. E) Quantification of pSTAT3/Total-STAT3 and Bcl2/Bax in Sham, SAH + AAV-control and SAH +AAV-THBS1 groups related to Figure 5L, n=4 per group, *** p < 0.001 by paired two-tailed Student's t test. F)

Quantification of pSTAT3/Total-STAT3 and Bcl2/Bax in Sham, SAH + igG, SAH + anti-CD47 and SAH + anti-THBS1 groups related to Figure 5M, n=4 per group, * $p < 0.05$, *** $p < 0.001$ by paired two-tailed Student's t test. Pooled data from at least 3 independent experiments.

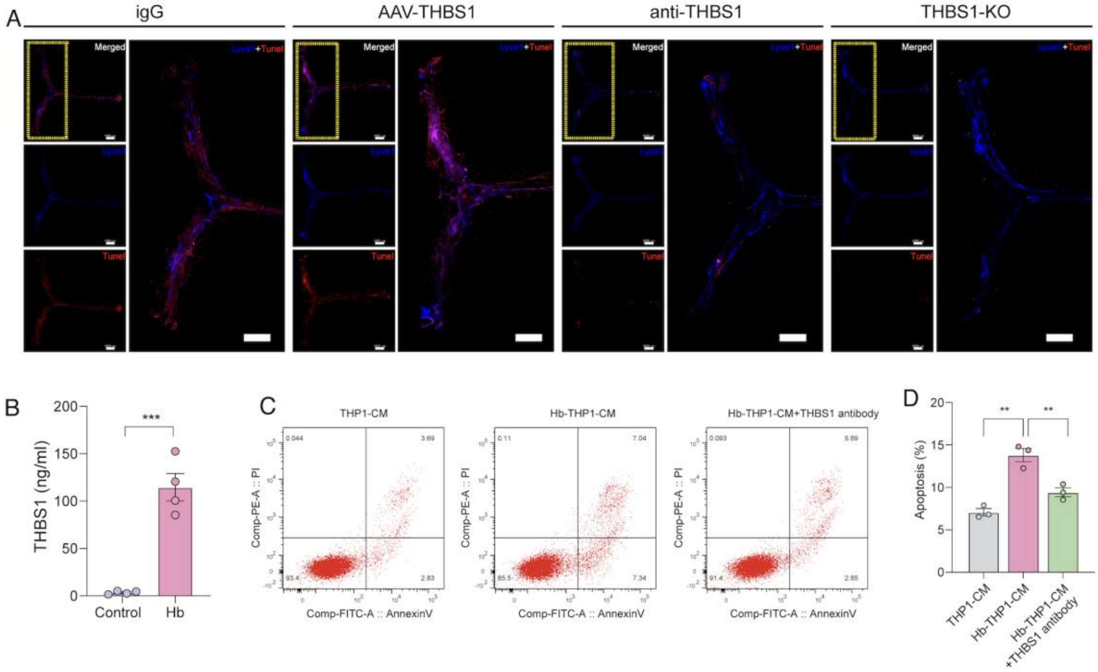

Supplementary Figure5 A) Tunel staining of mLECs from SAH and THBS1-manupelated mice. B) Quantification of THBS1 between THP1-CM without and with hemoglobin (150μM) treatment by Elisa assay, n=4 per group, *** $p < 0.001$ versus Control by Student's t test. C) Flow cytometry analysis of Annexin V and PI stained cells. D) Quantification of the percentage of Annexin V and PI double positive LECs, n=4 per group, ** $p < 0.01$ by paired two-tailed Student's t test.

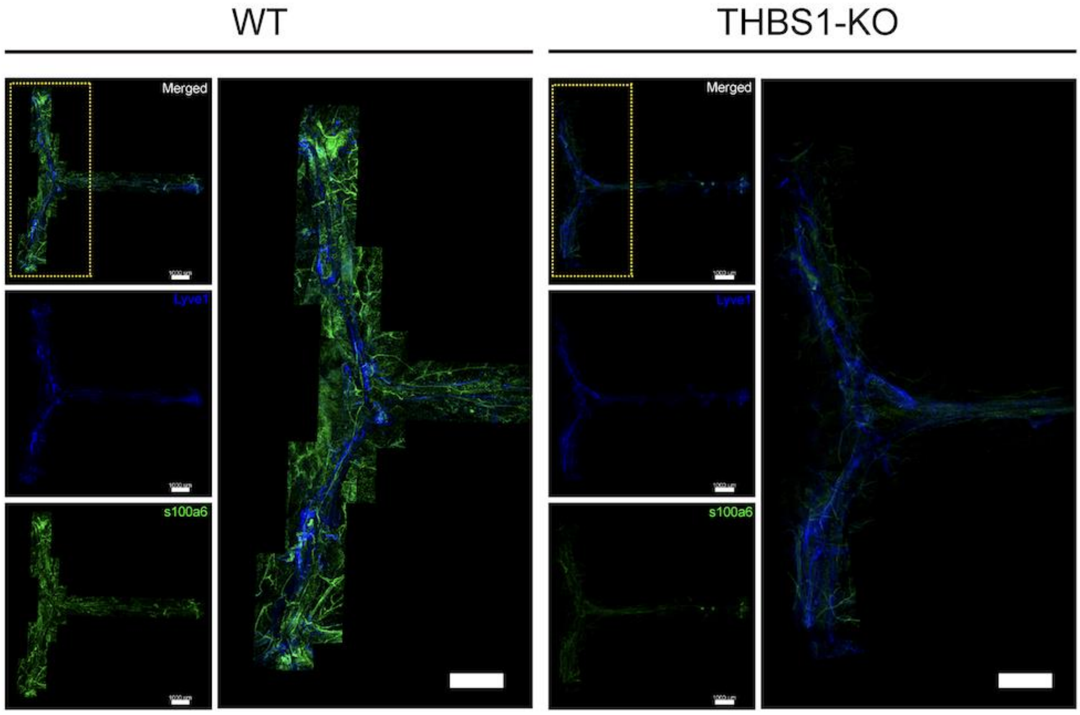

Supplementary Figure 6 Representative confocal images of mLVs region of WT and THBS1-KO mice after SAH 24h group. Enlarged view of selected region in Merged photo (yellow dotted box) are listed on the right in each group. Lyve1(blue) and S100α6 (green). Scale bar: 1000 μm for the holistic view.

Supplementary Table 1: Clinical data of SAH and NPH individuals

ID 1-48 were SAH individuals, and 49-64 were NPH individuals

ID	Age	Gender	THBS1 (ng/ml)	THBS2 (ng/ml)	THBS4 (ng/ml)	S100A6 (ng/ml)	mRS score	Unfavorable mRS (3-5)
**1**	59	femal	205.89	36.60	35.55	45.66	1	no
**2**	71	femal	419.14	36.09	13.46	47.75	5	yes
**3**	57	femal	953.39	45.74	291.92	48.05	1	no
**4**	68	male	603.68	37.95	18.46	46.30	5	yes
**5**	57	femal	632.50	46.64	39.65	46.67	2	no
**6**	48	femal	106.95	43.27	13.40	44.42	1	no
**7**	51	femal	337.68	40.39	281.73	46.20	1	no
**8**	41	male	363.02	41.89	16.66	46.69	2	no
**9**	52	femal	1554.87	29.85	18.39	56.83	5	yes
**10**	57	femal	85.92	44.07	302.64	46.63	1	no
**11**	74	male	800.28	41.78	81.09	49.35	5	yes
**12**	58	femal	695.68	45.75	578.35	52.05	4	yes
**13**	66	male	272.72	43.92	426.51	48.35	1	no
**14**	73	femal	459.73	46.75	316.95	49.75	5	yes
**15**	74	male	1000.28	42.59	469.76	54.35	5	yes
**16**	64	femal	95.65	45.01	514.66	48.85	1	no
**17**	51	femal	806.57	36.97	495.19	50.15	3	yes
**18**	39	male	113.13	45.13	419.82	47.05	4	yes
**19**	62	femal	1703.98	32.07	14.41	58.71	5	yes
**20**	75	male	197.44	41.62	13.33	47.75	4	no
**21**	64	femal	1421.66	43.22	555.02	51.65	5	yes
**22**	55	male	1218.35	44.92	488.50	47.84	1	no
**23**	57	male	1566.85	15.06	210.28	46.71	5	yes
**24**	43	femal	207.59	39.13	62.04	47.00	3	yes
**25**	48	male	66.56	25.47	22.44	45.43	4	yes
**26**	54	male	389.33	49.24	476.18	46.32	1	no
**27**	66	femal	666.16	42.29	452.73	45.36	1	no
**28**	18	femal	1628.56	43.68	501.79	46.06	5	yes
**29**	60	male	1266.69	44.18	508.49	45.51	4	yes
**30**	48	male	691.68	12.96	122.10	44.26	5	yes
**31**	85	femal	1576.92	38.31	252.19	46.22	5	yes
**32**	70	femal	546.64	40.09	455.37	47.75	5	yes
**33**	60	male	91.87	39.19	411.70	49.55	1	no
**34**	59	male	1446.70	15.28	496.74	45.56	2	no
**35**	67	male	151.16	40.39	561.15	46.90	5	yes
**36**	79	femal	457.09	43.88	593.62	49.45	6	yes
**37**	69	femal	652.54	43.05	553.11	45.29	4	yes

**38**	59	femal	937.09	42.70	462.82	46.91	1	no
**39**	71	femal	272.62	37.61	433.36	40.42	1	no
**40**	49	male	491.76	38.66	371.40	52.45	2	no
**41**	65	male	287.09	39.78	337.28	51.35	4	yes
**42**	54	male	549.19	42.61	524.54	45.49	1	no
**43**	54	male	485.96	41.73	547.58	45.49	2	no
**44**	51	male	201.29	37.63	265.96	44.85	1	no
**45**	51	femal	242.87	46.67	529.76	45.59	1	no
**46**	62	femal	91.97	40.91	473.72	45.30	5	yes
**47**	54	femal	440.19	11.81	52.63	46.76	1	no
**48**	66	male	621.58	43.62	81.89	46.37	6	yes
**49**	76	male	20.69	25.27	121.05	10.14		
**50**	68	male	17.80	30.38	149.91	9.58		
**51**	56	femal	24.80	23.45	187.11	9.27		
**52**	73	male	22.10	30.99	153.25	8.97		
**53**	53	femal	22.32	33.39	127.70	8.30		
**54**	61	femal	22.08	30.66	110.17	7.51		
**55**	59	femal	15.85	31.92	282.83	8.02		
**56**	71	male	32.58	26.69	89.92	10.69		
**57**	74	femal	24.29	19.38	46.63	10.94		
**58**	65	male	21.27	21.41	54.83	10.91		
**59**	69	male	19.97	26.37	113.03	10.26		
**60**	58	femal	22.30	27.90	89.48	10.22		
**61**	62	male	20.13	21.28	193.73	11.24		
**62**	74	femal	23.88	24.46	249.44	9.27		
**63**	79	femal	24.30	26.36	179.15	9.88		
**64**	58	male	23.27	24.98	68.27	10.23		

Supplementary Table 2: Mortality of animals

Group	Mortality
**SC-RNA and ST seq**	
Sham	0% (0/11)
SAH (24h, 72h)	15.4% (4/26)
**Immunofluorescence**	
**Meninges (fluorescent tracer)**	
Sham	0% (0/4)
SAH (24h,72h)	11.1% (1/9)
Sham+pbs	0% (0/4)
Sham+rTHBS1	0% (0/4)
**24h**	
Sham	0% (0/4)
SAH+WT	20% (1/5)
SAH+THBS1-KO	0% (0/4)
Sham	0% (0/4)
SAH+igG	20% (1/5)
SAH+anti-CD47	20% (1/5)
SAH+anti-THBS1	0% (0/4)
**72h**	
Sham	0% (0/4)
SAH+AAV-Control	20% (1/5)
SAH+AAV-THBS1	33.3% (2/6)
**Meninges (apoptosis)**	
**24h**	
SAH	20% (1/5)
SAH+AAV-THBS1	20% (1/5)
SAH+anti-THBS1	0% (0/4)
SAH+THBS1-KO	20% (1/5)
**Meninges (s100α6)**	
Sham	0% (0/4)
SAH	33.3% (2/6)
SAH+THBS1 KO	20% (1/5)
**dCLNs**	
Sham	0% (0/10)
SAH (3h,6h,12h,24h, 72h)	18.4% (9/49)
Sham+pbs	0% (0/8)
Sham+rTHBS1	0% (0/8)
**24h**	
Sham	0% (0/8)
SAH+WT	20% (2/10)
SAH+THBS1-KO	11.1% (1/9)
Sham	0% (0/8)
SAH+igG	27.3% (3/11)
SAH+anti-CD47	20% (2/10)

SAH+anti-THBS1	11.1% (1/9)
**72h**	
Sham	0% (0/8)
SAH+AAV-Control	20% (2/10)
SAH+AAV-THBS1	33.3% (4/12)
**Flow cytometric**	
Sham	0% (0/8)
SAH (24h, 72h)	11.1% (1/9)
Sham+pbs	0% (0/8)
Sham+rTHBS1	0% (0/8)
**24h**	
Sham	0% (0/8)
SAH+WT	20% (1/5)
SAH+THBS1-KO	0% (0/4)
Sham	0% (0/8)
SAH+igG	33.3% (2/6)
SAH+anti-CD47	20% (1/5)
SAH+anti-THBS1	0% (0/4)
**72h**	
Sham	0% (0/8)
SAH+AAV-Control	20% (1/5)
SAH+AAV-THBS1	33.3% (2/6)
**Western blot**	
primary mLEC culture	25 (P0-P1)
Sham	0% (0/12)
SAH (24h, 72h)	17.2% (5/29)
**24h**	
Sham	0% (0/12)
SAH+WT	14.3% (2/14)
SAH+THBS1-KO	7.7% (1/13)
Sham	0% (0/12)
SAH+igG	14.3% (2/14)
SAH+anti-CD47	14.3% (2/14)
SAH+anti-THBS1	7.7% (1/13)
**72h**	
Sham	0% (0/12)
SAH+AAV-Control	20% (3/15)
SAH+AAV-THBS1	25% (4/16)
**Total**	
Sham	0% (0/210)
SAH	17.6% (69/391)
